# Medical Waste Disposal Method Selection Based on a Hierarchical Decision Model with Intuitionistic Fuzzy Relations

**DOI:** 10.3390/ijerph13090896

**Published:** 2016-09-09

**Authors:** Wuyong Qian, Zhou-Jing Wang, Kevin W. Li

**Affiliations:** 1 School of Business, Jiangnan University, Wuxi 214122, China; qianyijiaemail@163.com; 2 School of Information, Zhejiang University of Finance & Economics, Hangzhou 310018, China; 3 Odette School of Business, University of Windsor, Windsor, ON N9B 3P4, Canada; kwli@uwindsor.ca; 4 College of Economics and Management, Fuzhou University, Fuzhou 350116, China

**Keywords:** medical waste disposal, multicriteria decision making, intuitionistic fuzzy values, operational laws, aggregation

## Abstract

Although medical waste usually accounts for a small fraction of urban municipal waste, its proper disposal has been a challenging issue as it often contains infectious, radioactive, or hazardous waste. This article proposes a two-level hierarchical multicriteria decision model to address medical waste disposal method selection (MWDMS), where disposal methods are assessed against different criteria as intuitionistic fuzzy preference relations and criteria weights are furnished as real values. This paper first introduces new operations for a special class of intuitionistic fuzzy values, whose membership and non-membership information is cross ratio based ]0, 1[-values. New score and accuracy functions are defined in order to develop a comparison approach for ]0, 1[-valued intuitionistic fuzzy numbers. A weighted geometric operator is then put forward to aggregate a collection of ]0, 1[-valued intuitionistic fuzzy values. Similar to Saaty’s 1–9 scale, this paper proposes a cross-ratio-based bipolar 0.1–0.9 scale to characterize pairwise comparison results. Subsequently, a two-level hierarchical structure is formulated to handle multicriteria decision problems with intuitionistic preference relations. Finally, the proposed decision framework is applied to MWDMS to illustrate its feasibility and effectiveness.

## 1. Introduction

Medical waste, in a broad sense, refers to solid or liquid waste generated when human beings or animals are diagnosed, treated, or immunized [1]. With the general public’s increasing awareness of environmental and health hazard of medical waste, its proper disposal and treatment has become a challenging issue around the globe [2]. Although medical waste usually accounts for a small fraction of urban municipal waste, its proper disposal has become a challenging issue as it often contains infectious, radioactive, or hazardous waste [1]. Due to the complex nature of medical waste as a result of different medical treatment procedures and facilities, multiple criteria have to be considered when different disposal methods are assessed. To minimize the risk to public health and environmental contamination, it is necessary to establish a systematic decision procedure to assist the evaluation of different alternatives and selection of the best choice [3]. This paper aims to propose a two-level hierarchical multicriteria decision model to address the medical waste disposal method selection (MWDMS) problem, where the criteria weights are furnished as real values and pairwise comparisons of different alternatives are provided as intuitionistic fuzzy judgment matrices.

For a complex multicriteria decision problem, pairwise comparison has been a popular and effective approach to eliciting a decision-maker’s (DM’s) preference [4]. In Saaty’s original monograph on the Analytic Hierarchy Process (AHP) [4], ratio-based pairwise ratings are provided in a 1–9 scale, resulting in a multiplicative preference relation. Another common representation of a DM’s ratings is based on a bipolar unit interval scale with a neutral value of 0.5, leading to a fuzzy preference relation [5] (also called a reciprocal preference relation [6,7]. To further characterize a DM’s hesitancy or vagueness in furnishing his/her membership judgments, Atanassov’s intuitionistic fuzzy sets emerge as a natural framework, where both membership and non-membership degrees are explicitly characterized [8]. Recent years have witnessed numerous applications of intuitionistic fuzzy sets to such areas as fuzzy clustering analysis [9], medical diagnosis [10], pattern recognition [11], and decision modeling [12], to name a few.

Xu [13] introduced operational laws for Atanassov’s intuitionistic fuzzy values (A-IFVs), and put forward the notion of intuitionistic preference relations (IPRs). Research has since been carried out for consistency of IPRs and decision modeling with IPRs [14], such as feasible-region-based consistency [15], mathematical-equation-based consistency [16,17,18], and priority elicitation methods [15,16,17]. Xu and Liao [19] presented a checking method for multiplicative consistency and put forward a framework for the intuitionistic fuzzy AHP. In the meantime, the and-like representable cross ratio (ALRCR) uninorm based functional equation was also extended to estimate unknown elements in incomplete IPRs [18,20]. The aforesaid research regards both membership and non-membership degrees of A-IFVs as ALRCR uninorm based values. The first part of this research is to establish new operational laws and a comparison method for ]0, 1[-valued A-IFVs.

In hierarchical mulcriteria decision making with preference relations, aggregation operators are often employed to obtain alternative priority based on pairwise comparison matrices. Numerous intuitionistic fuzzy aggregation operators have been developed and applied to decision making in the literature. For instance, based on operational laws of A-IFVs, Xu and Yager [21] put forward geometric aggregation operators for A-IFVs. Based on Einstein *t*-norm and *t*-conorm, Wang and Liu [22] developed some intuitionistic fuzzy Einstein aggregation operators. These aggregation operators treat membership and non-membership information differently by applying distinct operational laws. Based on the new operational laws proposed in this article, a new weighted geometric aggregation operator is developed for aggregating A-IFVs, where the same operational laws are applied to both membership and non-membership values.

In the process of pairwise comparison, a bounded bipolar scale is often needed to guide DMs to elicit their ratings [23]. Saaty’s 1–9 scale, as an example, is used in the classical AHP, in which a neutral value of 1 separates the preferred ratings from the non-preferred ones, and the values 1/9 and 9 are the lower and upper bounds. To better apply ]0, 1[-valued A-IFVs to decision making, this article develops a comparable bounded cross ratio based bipolar 0.1–0.9 scale for DMs to provide their evaluations as intuitionistic fuzzy judgments. A two-level hierarchical multicriteria decision model with IPRs under this scale is then developed for solving MWDMS problems.

The remainder of this paper is structured as follows. Section 2 reviews the notion of A-IFVs and the existing operations on A-IFVs. Section 3 puts forward new operations on A-IFVs and score and accuracy functions are defined to establish a comparison approach for ]0, 1[-valued A-IFVs. Section 4 develops an intuitionistic cross ratio weighted geometric (ICRWG) aggregation operator to aggregate ]0, 1[-valued A-IFVs and formulates a two-level hierarchical multicriteria decision procedure with IPRs. A MWDMS case study is then presented in Section 5 to illustrate how to apply the proposed decision framework in practice. Concluding remarks are furnished in Section 6.

## 2. Preliminaries

Let Z be a set of objects, a fuzzy set introduced by Zadeh [24] is defined as $A=\left\{\left(z,\mu_{A}(z)\right),z\in Z\right\}$, where $\mu_{A}:Z\to [0,1]$ is a membership mapping, and $\mu_{A}(z)$ gives the membership degree of element z belonging to *A*.

A fuzzy set only considers the membership information and implicitly assumes that non-membership is the complement of membership. To better depict vagueness and hesitancy, Atanassov [8] introduced an intuitionistic fuzzy set as

(1) $$B=\left\{\left(z,\xi_{B}(z),\eta_{B}(z)\right),z\in Z\right\}$$

where $\xi_{B}(z)$ and $\eta_{B}(z)$ are the membership and non-membership degrees of element z to *A*, respectively, and satisfy

(2) $$0\leq \xi_{B}(z),\eta_{B}(z)\leq 1,\xi_{B}(z)+\eta_{B}(z)\leq 1\;\forall z\in Z.$$

The pair $\left(\xi_{B}(z),\eta_{B}(z)\right)$ is called an A-IFV [13] and its hesitancy is measured by $1-\xi_{B}(z)-\eta_{B}(z)$. For notational convenience, A-IFV $\left(\xi_{B}(z),\eta_{B}(z)\right)$ is denoted by (ξ,η) for an Atanassov’s intuitionistic fuzzy set B and a given z, where 0≤ξ,η≤1 and ξ+η≤1.

For three A-IFVs $\tilde{\alpha }=(\xi ,\eta )$, ${\tilde{\alpha }}_{1}=(\xi_{1},\eta_{1})$ and ${\tilde{\alpha }}_{2}=(\xi_{2},\eta_{2})$, Xu [13] and Xu and Yager [21] defined the following basic operations:

1. ${\tilde{\alpha }}^{C}=(\eta ,\xi )$, i.e., ${\tilde{\alpha }}^{C}=(\eta ,\xi )$ is the complement of $\tilde{\alpha }=(\eta ,\xi )$;
2. ${\tilde{\alpha }}_{1}\wedge {\tilde{\alpha }}_{2}=\left(\min\left\{\xi_{1},\xi_{2}\right\},\max\left\{\eta_{1},\eta_{2}\right\}\right)$;
3. ${\tilde{\alpha }}_{1}\vee {\tilde{\alpha }}_{2}=\left(\max\left\{\xi_{1},\xi_{2}\right\},\min\left\{\eta_{1},\eta_{2}\right\}\right)$;
4. ${\tilde{\alpha }}_{1}+{\tilde{\alpha }}_{2}=\left(\xi_{1}+\xi_{2}-\xi_{1}\xi_{2},\eta_{1}\eta_{2}\right)$;
5. ${\tilde{\alpha }}_{1}\times {\tilde{\alpha }}_{2}=\left(\xi_{1}\xi_{2},\eta_{1}+\eta_{2}-\eta_{1}\eta_{2}\right)$;
6. $\lambda \tilde{\alpha }=\left(1-{\left(1-\xi \right)}^{\lambda },\eta^{\lambda }\right)$, where λ>0;
7. ${\left(\tilde{\alpha }\right)}^{\lambda }=\left(\xi^{\lambda },1-{\left(1-\eta \right)}^{\lambda }\right)$, where λ>0.

A-IFVs have been widely applied to decision modeling. Xu [13] employed them to introduce the concept of IPRs, in which preferences over decision alternative pairs are expressed as A-IFVs.

Let $X=\{x_{1},x_{2},\ldots ,x_{n}\}$ be a set of alternatives. An IPR on X is characterized by a comparison matrix $\tilde{R}={\left({\tilde{r}}_{ij}\right)}_{n\;\times \;n}$ satisfying

(3) $${\tilde{r}}_{ij}=(\xi_{ij},\eta_{ij}),{\tilde{r}}_{ii}=(0.5,0.5),{\tilde{r}}_{ji}={\tilde{r}}_{ij}{}^{C}\;\forall i,j=1,2,\ldots ,n$$

where ${\tilde{r}}_{ij}$ is an A-IFV, and $\xi_{ij}$ indicates the membership value to which $x_{i}$ is preferred to $x_{j}$, $\eta_{ij}$ shows the non-membership value to which $x_{i}$ is not preferred to $x_{j}$. In particular, if ${\tilde{r}}_{ij}=(0.5,0.5)$, alternatives $x_{i}$ and $x_{j}$ are indifferent for the DM.

## 3. New Operations and Comparison of Atanassov’s Intuitionistic Fuzzy Values

This section first establishes new operational laws for A-IFVs and presents their properties. Then, new score, hesitation margin and accuracy functions are defined for ]0, 1[-valued A-IFVs, followed by an approach to comparing ]0, 1[-valued A-IFVs.

**Definition 1.**  *Let* $\tilde{\alpha }=(\xi ,\eta )$, ${\tilde{\alpha }}_{1}=(\xi_{1},\eta_{1})$ and ${\tilde{\alpha }}_{2}=(\xi_{2},\eta_{2})$ *be three A-IFVs with* $0<\xi ,\eta ,\xi_{1_{i}},\eta_{1},\xi_{2_{i}},\eta_{2}<1$, *then*

(i) ${\tilde{\alpha }}_{1}⊕{\tilde{\alpha }}_{2}=\left(\frac{\xi_{1}+\xi_{2}-2\xi_{1}\xi_{2}}{1-\xi_{1}\xi_{2}},\frac{\eta_{1}\eta_{2}}{\eta_{1}+\eta_{2}-\eta_{1}\eta_{2}}\right)$*;*
(ii) ${\tilde{\alpha }}_{1}⊗{\tilde{\alpha }}_{2}=\left(\frac{\xi_{1}\xi_{2}}{\xi_{1}\xi_{2}+(1-\xi_{1})(1-\xi_{2})},\frac{\eta_{1}\eta_{1}}{\eta_{1}\eta_{2}+(1-\eta_{1})(1-\eta_{2})}\right)$*;*
(iii) $\lambda \tilde{\alpha }=\left(\frac{\lambda \xi }{\lambda \xi +(1-\xi )},\frac{\eta }{\eta +\lambda (1-\eta )}\right)$*, where* λ>0*;*
(iv) ${\left(\tilde{\alpha }\right)}^{\lambda }=\left(\frac{{\left(\xi \right)}^{\lambda }}{{\left(\xi \right)}^{\lambda }+{\left(1-\xi \right)}^{\lambda }},\frac{{\left(\eta \right)}^{\lambda }}{{\left(\eta \right)}^{\lambda }+{\left(1-\eta \right)}^{\lambda }}\right)$*, where* λ>0.

It can be verified that the aforesaid four basic operations always yield ]0, 1[-valued A-IFVs. These operational laws will be employed in the remainder of the paper.

(ii) in Definition 1 indicates that the multiplication operation “⊗” is based on the ALRCR uninorm $\frac{xy}{xy+(1-x)(1-y)}$ proposed by Chiclana et al. [6], which can be equivalently rewritten as $\frac{\frac{x}{\left(1-x\right)}\frac{y}{\left(1-y\right)}}{1+\frac{x}{\left(1-x\right)}\frac{y}{\left(1-y\right)}}$. Moreover, the membership and non-membership values of ${\tilde{\alpha }}_{1}$ and ${\tilde{\alpha }}_{2}$ are treated by the same operational rule.

It should be noted that the multiplication operation “⊗” differs from the operation “${⊗}_{e}$” proposed by Wang and Liu [22], where the membership and non-membership values are, respectively, treated by using ALRCR uninorm and Einstein *t*-conorm $\frac{x+y}{1+xy}$, i.e., ${\tilde{\alpha }}_{1}{⊗}_{e}{\tilde{\alpha }}_{2}=(\xi_{1},\eta_{1}){⊗}_{e}(\xi_{2},\eta_{2})=\left(\frac{\xi_{1}\xi_{2}}{\xi_{1}\xi_{2}+(1-\xi_{1})(1-\xi_{2})},\frac{\eta_{1}+\eta_{2}}{1+\eta_{1}\eta_{2}}\right)$.

One can easily verify that $\frac{\eta_{1}\eta_{2}}{\eta_{1}+\eta_{2}-\eta_{1}\eta_{2}}=1-\frac{\left(1-\eta_{1})+(1-\eta_{1})-2(1-\eta_{1})(1-\eta_{2}\right)}{1-(1-\eta_{1})(1-\eta_{2})}$. Thus, the addition operation “⊕” is based on the or-like representable cross ratio *t*-conorm: $\frac{x+y-2xy}{1-xy}$, which can be equivalently expressed as $\frac{\frac{x}{\left(1-x\right)}+\frac{y}{\left(1-y\right)}}{1+\frac{x}{\left(1-x\right)}+\frac{y}{\left(1-y\right)}}$.

Based on Definition 1, one can prove that these operations possess the following properties:

**Theorem 1.**  *Let* ${\tilde{\alpha }}_{1}=(\eta_{1},\xi_{1})$ *and* ${\tilde{\alpha }}_{2}=(\eta_{2},\xi_{2})$ *be two A-IFVs with* $0<\xi_{1},\eta_{1},\xi_{2},\eta_{2}<1$, *then*

(1) ${\tilde{\alpha }}_{1}⊕{\tilde{\alpha }}_{2}={\tilde{\alpha }}_{2}⊕{\tilde{\alpha }}_{1}$*;*
(2) ${\tilde{\alpha }}_{1}⊗{\tilde{\alpha }}_{2}={\tilde{\alpha }}_{2}⊗{\tilde{\alpha }}_{1}$*;*
(3) $\lambda ({\tilde{\alpha }}_{1}⊕{\tilde{\alpha }}_{2})=\lambda {\tilde{\alpha }}_{1}⊕\lambda {\tilde{\alpha }}_{2}$*, where* λ>0*;*
(4) ${\left({\tilde{\alpha }}_{1}⊗{\tilde{\alpha }}_{2}\right)}^{\lambda }={\left({\tilde{\alpha }}_{1}\right)}^{\lambda }⊗{\left({\tilde{\alpha }}_{2}\right)}^{\lambda }$*, where* λ>0*;*
(5) $\lambda_{1}{\tilde{\alpha }}_{1}⊕\lambda_{2}{\tilde{\alpha }}_{1}=(\lambda_{1}+\lambda_{2}){\tilde{\alpha }}_{1}$*, where* $\lambda_{1},\lambda_{2}>0$*;*
(6) ${\left({\tilde{\alpha }}_{1}\right)}^{\lambda_{1}}⊗{\left({\tilde{\alpha }}_{1}\right)}^{\lambda_{2}}={\left({\tilde{\alpha }}_{1}\right)}^{\lambda_{1}+\lambda_{2}}$*, where* $\lambda_{1},\lambda_{2}>0$.

If two A-IFVs ${\tilde{\alpha }}_{1}=(\xi_{1},\eta_{1})$ and ${\tilde{\alpha }}_{2}=(\xi_{2},\eta_{2})$ are reduced to ordinal fuzzy values, one can confirm that ${\tilde{\alpha }}_{1}⊕{\tilde{\alpha }}_{2}$, ${\tilde{\alpha }}_{1}⊗{\tilde{\alpha }}_{2}$, $\lambda {\tilde{\alpha }}_{1}$, and ${\left({\tilde{\alpha }}_{1}\right)}^{\lambda }$ are all reduced to ordinary fuzzy values, where λ is a positive real value.

By mathematical induction, one can easily prove the following result.

**Theorem 2.**  *Let* $\tilde{\alpha }=(\xi ,\eta )$ *be an A-IFV with* 0<ξ,η<1*, then for any positive integer n, we have* $n\tilde{\alpha }=\underset{n}{\underset{︸}{\tilde{\alpha }⊕\ldots ⊕\tilde{\alpha }}},{\left(\tilde{\alpha }\right)}^{n}=\underset{n}{\underset{︸}{\tilde{\alpha }⊗\ldots ⊗\tilde{\alpha }}}$*.*

Wang [17] introduced an intuitionistic fuzzy geometric index to measure the amount of an A-IFV from the viewpoint of ratio-based intuitionistic fuzzy judgments. Similarly, we define the following score function for A-IFVs.

**Definition 2.**  *Let* $\tilde{\alpha }=(\xi ,\eta )$ *be an A-IFV with* 0<ξ,η<1, *the score of* $\tilde{\alpha }$ *is defined as*

(4) $$S(\tilde{\alpha })=\frac{\sqrt{\xi (1-\eta )}}{\sqrt{\xi (1-\eta )}+\sqrt{(1-\xi )\eta }}$$

It is obvious that $0<S(\tilde{\alpha })<1$. If ξ>0.5, we have $S(\tilde{\alpha })>0.5$; if η>0.5, one can get $S(\tilde{\alpha })<0.5$. In particular, if $\tilde{\alpha }=(0.5,0.5)$, then $S(\tilde{\alpha })=0.5$. If ξ→1, then $S(\tilde{\alpha })\to 1$; if η→1, then $S(\tilde{\alpha })\to 0$. The larger the value of $S(\tilde{\alpha })$, the bigger the A-IFV $\tilde{\alpha }$. For two A-IFVs ${\tilde{\alpha }}_{1}=(\xi_{1},\eta_{1})$ and ${\tilde{\alpha }}_{2}=(\xi_{2},\eta_{2})$ with $0<\xi_{1},\eta_{1},\xi_{2},\eta_{2}<1$, if $\xi_{1}\geq \xi_{2}$ and $\eta_{1}\leq \eta_{2}$, then we have $S({\tilde{\alpha }}_{1})\geq S({\tilde{\alpha }}_{2})$.

**Definition 3.**  [17] *Let* $\tilde{\alpha }=(\xi ,\eta )$ *be an A-IFV with* 0<ξ,η<1*, then its hesitation margin is defined as*

(5) $$\pi (\tilde{\alpha })=\frac{1-\xi -\eta }{\left(1-\xi )(1-\eta \right)}$$

Equation (5) can be rewritten as:

(6) $$\pi (\tilde{\alpha })=1-\frac{\xi \eta }{\left(1-\xi )(1-\eta \right)}$$

Since 0<ξ,η<1 and ξ+η≤1, we have 0≤1−ξ−η<1−ξ−η+ξη=(1−ξ)(1−η). Thus, $0\leq \pi (\tilde{\alpha })<1$. If $\frac{\xi \eta }{\left(1-\xi )(1-\eta \right)}=1$, then $\pi (\tilde{\alpha })=0$, and $\tilde{\alpha }$ is reduced to an ordinary fuzzy value, i.e., ξ+η=1. If $\frac{\xi \eta }{\left(1-\xi )(1-\eta \right)}\to 0$, then $\pi (\tilde{\alpha })\to 1$ and $\tilde{\alpha }$ is extremely hesitant. If $\frac{\xi \eta }{\left(1-\xi )(1-\eta \right)}\to 1$, then $\pi (\tilde{\alpha })\to 0$ and $\tilde{\alpha }$ has little hesitation and is highly accurate.

Based on Equation (5) or Equation (6), the following accuracy function is introduced for A-IFVs.

**Definition 4.**  *Let* $\tilde{\alpha }=(\xi ,\eta )$ *be an A-IFV with* 0<ξ,η<1*, the accuracy of* $\tilde{\alpha }$ *is measured by*

(7) $$H(\tilde{\alpha })=\frac{\xi \eta }{\left(1-\xi )(1-\eta \right)}$$

Obviously, $H(\tilde{\alpha })=1-\pi (\tilde{\alpha })$. Thus, $0<H(\tilde{\alpha })\leq 1$. The larger the $H(\tilde{\alpha })$, the higher the accuracy of the A-IFV $\tilde{\alpha }$. In particular, if $H(\tilde{\alpha })=1$, then $\tilde{\alpha }$ is reduced to an ordinary fuzzy value and ξ+η=1.

Based on the aforesaid analysis, a comparison approach is introduced to rank ]0, 1[-valued A-IFVs.

**Definition 5.**  *Let* ${\tilde{\alpha }}_{1}=(\xi_{1},\eta_{1})$ *and* ${\tilde{\alpha }}_{2}=(\xi_{2},\eta_{2})$ *be two A-IFVs with* $0<\xi_{1},\eta_{1},\xi_{2},\eta_{2}<1$*, then*

*If* $S({\tilde{\alpha }}_{1})>S({\tilde{\alpha }}_{2})$*, then* ${\tilde{\alpha }}_{1}>{\tilde{\alpha }}_{2}$*, indicating* ${\tilde{\alpha }}_{1}$ *is larger than* ${\tilde{\alpha }}_{2}$*;*

*If* $S({\tilde{\alpha }}_{1})=S({\tilde{\alpha }}_{2})$*, then*

*If* $H({\tilde{\alpha }}_{1})>H({\tilde{\alpha }}_{2})$*, then* ${\tilde{\alpha }}_{1}>{\tilde{\alpha }}_{2}$*;*
*If* $H({\tilde{\alpha }}_{1})=H({\tilde{\alpha }}_{2})$*, then* ${\tilde{\alpha }}_{1}={\tilde{\alpha }}_{2}$*.*

By (4), (6) and Definition 5, we have ${\tilde{\alpha }}_{1}={\tilde{\alpha }}_{2}$ if and only if $\xi_{1}=\xi_{2}$ and $\eta_{1}=\eta_{2}$. Denote ${\tilde{\alpha }}_{1}\geq {\tilde{\alpha }}_{2}$ if and only if ${\tilde{\alpha }}_{1}>{\tilde{\alpha }}_{2}$ or ${\tilde{\alpha }}_{1}={\tilde{\alpha }}_{2}$.

**Theorem 3.**  *Let* ${\tilde{\alpha }}_{1}=(\xi_{1},\eta_{1})$ *and* ${\tilde{\alpha }}_{2}=(\xi_{2},\eta_{2})$ *be two A-IFVs with* $0<\xi_{1},\eta_{1},\xi_{2},\eta_{2}<1$. *If* $\xi_{1}\geq \xi_{2}$ *and* $\eta_{1}\leq \eta_{2}$*, then* ${\tilde{\alpha }}_{1}\geq {\tilde{\alpha }}_{2}$.

**Proof.**  Since $\xi_{1}\geq \xi_{2}$ and $\eta_{1}\leq \eta_{2}$, we have

$$\begin{array}{l} \frac{1}{\xi_{1}}\leq \frac{1}{\xi_{2}},\frac{1}{\eta_{1}}\geq \frac{1}{\eta_{2}}\;\Rightarrow \;\frac{1}{\xi_{1}}-1\leq \frac{1}{\xi_{2}}-1,\frac{1}{\eta_{1}}-1\geq \frac{1}{\eta_{2}}-1\;\Rightarrow \\ \left(\frac{1}{\xi_{1}}-1\right)/\left(\frac{1}{\eta_{1}}-1\right)\leq \left(\frac{1}{\xi_{2}}-1\right)/\left(\frac{1}{\eta_{2}}-1\right)\Rightarrow \frac{(1-\xi_{1})\eta_{1}}{\xi_{1}(1-\eta_{1})}\leq \frac{(1-\xi_{2})\eta_{2}}{\xi_{2}(1-\eta_{2})}\;\Rightarrow \\ 1+\sqrt{\frac{(1-\xi_{1})\eta_{1}}{\xi_{1}(1-\eta_{1})}}\leq 1+\sqrt{\frac{(1-\xi_{2})\eta_{2}}{\xi_{2}(1-\eta_{2})}}\;\Rightarrow 1/\left(1+\sqrt{\frac{(1-\xi_{1})\eta_{1}}{\xi_{1}(1-\eta_{1})}}\right)\geq 1/\left(1+\sqrt{\frac{(1-\xi_{2})\eta_{2}}{\xi_{2}(1-\eta_{2})}}\right)\\ \Rightarrow \;S({\tilde{\alpha }}_{1})=\frac{\sqrt{\xi_{1}(1-\eta_{1})}}{\sqrt{\xi_{1}(1-\eta_{1})}+\sqrt{(1-\xi_{1})\eta_{1}}}\geq \frac{\sqrt{\xi_{2}(1-\eta_{2})}}{\sqrt{\xi_{2}(1-\eta_{2})}+\sqrt{(1-\xi_{2})\eta_{2}}}=S({\tilde{\alpha }}_{2}) \end{array}$$

If $S({\tilde{\alpha }}_{1})>S({\tilde{\alpha }}_{2})$, then ${\tilde{\alpha }}_{1}>{\tilde{\alpha }}_{2}$.

If $S({\tilde{\alpha }}_{1})=S({\tilde{\alpha }}_{2})$, then $\frac{(1-\xi_{1})\eta_{1}}{\xi_{1}(1-\eta_{1})}=\frac{(1-\xi_{2})\eta_{2}}{\xi_{2}(1-\eta_{2})}$. It follows that $\frac{\eta_{1}}{\left(1-\eta_{1}\right)}=\frac{\xi_{1}(1-\xi_{2})\eta_{2}}{\left(1-\xi_{1})\xi_{2}(1-\eta_{2}\right)}$.

As per (7), one can obtain

$$H({\tilde{\alpha }}_{1})=\frac{\xi_{1}\eta_{1}}{\left(1-\xi_{1})(1-\eta_{1}\right)}={\left(\frac{\xi_{1}}{1-\xi_{1}}\right)}^{2}\frac{(1-\xi_{2})\eta_{2}}{\xi_{2}(1-\eta_{2})}\geq {\left(\frac{\xi_{2}}{1-\xi_{2}}\right)}^{2}\frac{(1-\xi_{2})\eta_{2}}{\xi_{2}(1-\eta_{2})}=\frac{\xi_{2}\eta_{2}}{\left(1-\xi_{2})(1-\eta_{2}\right)}=H({\tilde{\alpha }}_{2}).$$

Therefore, ${\tilde{\alpha }}_{1}\geq {\tilde{\alpha }}_{2}$.

## 4. A Hierarchical Multicriteria Decision Model with Intuitionistic Preference Relations

This section first develops an ICRWG aggregation operator to aggregate ]0, 1[-valued A-IFVs and, then, presents a two-level hierarchical multicriteria decision model with IPRs.

### 4.1. An Intuitionistic Cross Ratio Weighted Geometric Operator

Let

(8) Θ={(ξ,η)|0<ξ,η<1,ξ+η≤1}

**Definition 6.**  *Let* ${\tilde{\alpha }}_{i}=(\xi_{i},\eta_{i})$ *(*i=1,2,…,m*)* *be m A-IFVs in* Θ*, if a mapping* $\mathrm{ICRWG}:\;\Theta^{m}\to \Theta$ *satisfies*

(9) $${\mathrm{ICRWG}}_{\omega }({\tilde{\alpha }}_{1},{\tilde{\alpha }}_{2},\ldots ,{\tilde{\alpha }}_{m})=\overset{m}{\underset{i\;=\;1}{⊗}}{\left({\tilde{\alpha }}_{i}\right)}^{\omega_{i}}$$

*where* $\omega ={\left(\omega_{1},\omega_{2},\ldots ,\omega_{m}\right)}^{T}$ *is a weight vector for A-IFVs* ${\tilde{\alpha }}_{i}$ *(*i=1,2,…,m*), with* $\omega_{i}\in [0,1]$ *(*i=1,2,…,m*) and* $\sum_{i\;=\;1}^{m}\omega_{i}=1$*, then it is called an ICRWG operator of dimension m*.

By mathematical induction, one can prove the following theorem.

**Theorem 4.**  *Let* ${\tilde{\alpha }}_{i}=(\xi_{i},\eta_{i})$ *(*i=1,2,…,m*) be m A-IFVs in* Θ*, then the aggregated value* ${\mathrm{ICRWG}}_{\omega }({\tilde{\alpha }}_{1},{\tilde{\alpha }}_{2},\ldots ,{\tilde{\alpha }}_{m})$ *defined by (9) is also an A-IFV in* Θ*, and*

(10) $${\mathrm{ICRWG}}_{\omega }({\tilde{\alpha }}_{1},{\tilde{\alpha }}_{2},\ldots ,{\tilde{\alpha }}_{m})=\left(\frac{\prod_{i\;=\;1}^{m}{\left(\xi_{i}\right)}^{\omega_{i}}}{\prod_{i\;=\;1}^{m}{\left(\xi_{i}\right)}^{\omega_{i}}+\prod_{i\;=\;1}^{m}{\left(1-\xi_{i}\right)}^{\omega_{i}}},\frac{\prod_{i\;=\;1}^{m}{\left(\eta_{i}\right)}^{\omega_{i}}}{\prod_{i\;=\;1}^{m}{\left(\eta_{i}\right)}^{\omega_{i}}+\prod_{i\;=\;1}^{m}{\left(1-\eta_{i}\right)}^{\omega_{i}}}\right)$$

In particular, if the A-IFVs ${\tilde{\alpha }}_{i}=(\xi_{i},\eta_{i})$ (i=1,2,…,m) are reduced to ordinary fuzzy values, i.e., $\xi_{i}+\eta_{i}=1$ for all i=1,2,…,m, then (10) is reduced to an ordinary fuzzy value as follows:

(11) $${\mathrm{ICRWG}}_{\omega }({\tilde{\alpha }}_{1},{\tilde{\alpha }}_{2},\ldots ,{\tilde{\alpha }}_{m})=\left(\frac{\prod_{i\;=\;1}^{m}{\left(\xi_{i}\right)}^{\omega_{i}}}{\prod_{i\;=\;1}^{m}{\left(\xi_{i}\right)}^{\omega_{i}}+\prod_{i\;=\;1}^{m}{\left(1-\xi_{i}\right)}^{\omega_{i}}},\frac{\prod_{i\;=\;1}^{m}{\left(1-\xi_{i}\right)}^{\omega_{i}}}{\prod_{i\;=\;1}^{m}{\left(\xi_{i}\right)}^{\omega_{i}}+\prod_{i\;=\;1}^{m}{\left(1-\xi_{i}\right)}^{\omega_{i}}}\right)$$

If $\omega ={\left(1/m,1/m,\ldots ,1/m\right)}^{T}$, then the ICRWG operator is reduced to the intuitionistic cross ratio weighted geometric mean (ICRWGM) operator:

(12) $$\begin{array}{l} \mathrm{ICRWGM}({\tilde{\alpha }}_{1},{\tilde{\alpha }}_{2},\ldots ,{\tilde{\alpha }}_{m})=\overset{m}{\underset{i\;=\;1}{⊗}}{\left({\tilde{\alpha }}_{i}\right)}^{1/m}\\ =\left(\frac{\sqrt[{m}]{\prod_{i\;=\;1}^{m}\xi_{i}}}{\sqrt[{m}]{\prod_{i\;=\;1}^{m}\xi_{i}}+\sqrt[{m}]{\prod_{i\;=\;1}^{m}\left(1-\xi_{i}\right)}},\frac{\sqrt[{m}]{\prod_{i\;=\;1}^{m}\eta_{i}}}{\sqrt[{m}]{\prod_{i\;=\;1}^{m}\eta_{i}}+\sqrt[{m}]{\prod_{i\;=\;1}^{m}\left(1-\eta_{i}\right)}}\right) \end{array}$$

**Example 1.**  *Let* ${\tilde{\alpha }}_{1}=(0.9,0.1)$*,* ${\tilde{\alpha }}_{2}=(0.9,0.1)$*,* ${\tilde{\alpha }}_{3}=(0.9,0.1)$ *and* ${\tilde{\alpha }}_{4}=(0.1,0.9)$ *be four A-IFVs, and* $\omega ={\left(0.25,0.25,0.25,0.25\right)}^{T}$ *be their weight vector, then by (12), one has* $\mathrm{ICRWGM}({\tilde{\alpha }}_{1},{\tilde{\alpha }}_{2},{\tilde{\alpha }}_{3},{\tilde{\alpha }}_{4})=\overset{4}{\underset{i\;=\;1}{⊗}}{\left({\tilde{\alpha }}_{i}\right)}^{1/4}=(0.75,0.25)$*.*

Xu and Yager [21] put forward an intuitionistic fuzzy weighted geometric (IFWG) operator as ${\mathrm{IFWG}}_{\omega }({\tilde{\alpha }}_{1},{\tilde{\alpha }}_{2},\ldots ,{\tilde{\alpha }}_{m})=\left(\prod_{i\;=\;1}^{m}{\left(\xi_{i}\right)}^{\omega_{i}},1-\prod_{i\;=\;1}^{m}{\left(1-\eta_{i}\right)}^{\omega_{i}}\right)$. Based on Einstein *t*-norm and *t*-conorm, Wang and Liu [22] developed an intuitionistic fuzzy Einstein weighted aggregation operator as

$$\begin{array}{l} {\mathrm{IFGA}}_{\omega }^{e}({\tilde{\alpha }}_{1},{\tilde{\alpha }}_{2},\ldots ,{\tilde{\alpha }}_{m})={\left({\tilde{\alpha }}_{1}\right)}^{\omega_{1}}{⊗}_{e}{\left({\tilde{\alpha }}_{2}\right)}^{\omega_{2}}{⊗}_{e}\ldots {⊗}_{e}{\left({\tilde{\alpha }}_{m}\right)}^{\omega_{m}}\\ =\left(\frac{2\prod_{i\;=\;1}^{m}{\left(\xi_{i}\right)}^{\omega_{i}}}{\prod_{i\;=\;1}^{m}{\left(2-\xi_{i}\right)}^{\omega_{i}}+\prod_{i\;=\;1}^{m}{\left(\xi_{i}\right)}^{\omega_{i}}},\frac{\prod_{i\;=\;1}^{m}{\left(1+\eta_{i}\right)}^{\omega_{i}}-\prod_{i\;=\;1}^{m}{\left(1-\eta_{i}\right)}^{\omega_{i}}}{\prod_{i\;=\;1}^{m}{\left(1+\eta_{i}\right)}^{\omega_{i}}+\prod_{i\;=\;1}^{m}{\left(1-\eta_{i}\right)}^{\omega_{i}}}\right) \end{array}$$

By using the same four A-IFVs in Example 1, one can obtain ${\mathrm{IFWG}}_{\omega }({\tilde{\alpha }}_{1},{\tilde{\alpha }}_{2},{\tilde{\alpha }}_{3},{\tilde{\alpha }}_{4})=$ (0.5196,0.4804) and ${\mathrm{IFGA}}_{\omega }^{e}({\tilde{\alpha }}_{1},{\tilde{\alpha }}_{2},{\tilde{\alpha }}_{3},{\tilde{\alpha }}_{4})=\left(0.5836,0.4164\right)$.

In this example, the four input A-IFVs are reduced to fuzzy numbers (as the membership and non-membership degrees add up to 1), where the first three values can be interpreted as 90% membership in support of the judgment and the last value represents 10% membership. It is apparent that the three aggregation operators qualitatively yield the same result in the sense of a higher membership degree than the corresponding non-membership degree. However, their discrimination powers differ: our aggregated value of (0.75, 0.25) has a proper gap between the membership and non-membership degrees to reflect the DM’s strength in his/her preference judgment. On the contrary, the other two aggregation operators yield much smaller gaps between the membership and non-membership degrees, indicating the DM’s much weaker preference strength. It is clear that the results from the existing aggregation operators by Xu and Yager [21] and Wang and Liu [22] are inconsistent with our intuition. This example justifies the introduction of the new operational laws, the associated aggregation operator and the following decision procedure.

It is easy to confirm that the proposed ICRWG operator is bounded, monotonic, and idempotent.

**Theorem 5.**  *(Boundedness). Let* ${\tilde{\alpha }}_{i}=(\xi_{i},\eta_{i})$ *(*i=1,2,…,m*)* *be m A-IFVs in* Θ*, then*

(13) $${\tilde{\alpha }}_{\max}\geq {\mathrm{ICRWG}}_{\omega }({\tilde{\alpha }}_{1},{\tilde{\alpha }}_{2},\ldots ,{\tilde{\alpha }}_{m})\geq {\tilde{\alpha }}_{\min}$$

*where* ${\tilde{\alpha }}_{\min}=\left(\underset{i}{\min}\{\xi_{i}\},\underset{i}{\max}\{\eta_{i}\}\right),{\tilde{\alpha }}_{\max}=\left(\underset{i}{\max}\{\xi_{i}\},\underset{i}{\min}\{\eta_{i}\}\right)$ *and the order relation of “* ≥*” is defined by the comparison method under Definition 5.*

**Theorem 6.**  *(Monotonicity). Let* ${\tilde{\alpha }}_{i}=(\xi_{i},\eta_{i})$ *(*i=1,2,…,m*) and* ${\tilde{\alpha }}_{i}^{\prime }=(\xi_{i}^{\prime },\eta_{i}^{\prime })$ *(*i=1,2,…,m*) be two collections of A-IFVs in* Θ*, if* ${\tilde{\alpha }}_{i}\geq {\tilde{\alpha }}_{i}^{\prime }$ *for all* i=1,2,…,m*, then*

(14) $${\mathrm{ICRWG}}_{\omega }({\tilde{\alpha }}_{1},{\tilde{\alpha }}_{2},\ldots ,{\tilde{\alpha }}_{m})\geq {\mathrm{ICRWG}}_{\omega }({\tilde{\alpha }}_{1}^{\prime },{\tilde{\alpha }}_{2}^{\prime },\ldots ,{\tilde{\alpha }}_{m}^{\prime })$$

**Proof.**  As ${\tilde{\alpha }}_{i}\geq {\tilde{\alpha }}_{i}^{\prime }$, we have

$S({\tilde{\alpha }}_{i})=\frac{\sqrt{\xi_{i}(1-\eta_{i})}}{\sqrt{\xi_{i}(1-\eta_{i})}+\sqrt{(1-\xi_{i})\eta_{i}}}>\frac{\sqrt{\xi_{i}^{\prime }(1-\eta_{i}^{\prime })}}{\sqrt{\xi_{i}^{\prime }(1-\eta_{i}^{\prime })}+\sqrt{(1-\xi_{i}^{\prime })\eta_{i}^{\prime }}}=S({\tilde{\alpha }}_{i}^{\prime })$ or

$S({\tilde{\alpha }}_{i})=S({\tilde{\alpha }}_{i}^{\prime })$ and $H({\tilde{\alpha }}_{i})=\frac{\xi_{i}\eta_{i}}{\left(1-\xi_{i})(1-\eta_{i}\right)}\geq \frac{\xi_{i}^{\prime }\eta_{i}^{\prime }}{\left(1-\xi_{i}^{\prime })(1-\eta_{i}^{\prime }\right)}=H({\tilde{\alpha }}_{i}^{\prime })$.

It follows that $\frac{(1-\xi_{i})\eta_{i}}{\xi_{i}(1-\eta_{i})}<\frac{(1-\xi_{i}^{\prime })\eta_{i}^{\prime }}{\xi_{i}^{\prime }(1-\eta_{i}^{\prime })}$ or

$$\frac{(1-\xi_{i})\eta_{i}}{\xi_{i}(1-\eta_{i})}=\frac{(1-\xi_{i})\eta_{i}^{\prime }}{\xi_{i}(1-\eta_{i}^{\prime })}\text{ and }\frac{\xi_{i}\eta_{i}}{\left(1-\xi_{i})(1-\eta_{i}\right)}\geq \frac{\xi_{i}^{\prime }\eta_{i}^{\prime }}{\left(1-\xi_{i}^{\prime })(1-\eta_{i}^{\prime }\right)}.$$

Thus, ${\prod_{i=1}^{m}\left(\frac{(1-\xi_{i})\eta_{i}}{\xi_{i}(1-\eta_{i})}\right)}^{\omega_{i}}<{\prod_{i=1}^{m}\left(\frac{(1-\xi_{i}^{\prime })\eta_{i}^{\prime }}{\xi_{i}^{\prime }(1-\eta_{i}^{\prime })}\right)}^{\omega_{i}}$ or

$${\prod_{i\;=\;1}^{m}\left(\frac{(1-\xi_{i})\eta_{i}}{\xi_{i}(1-\eta_{i})}\right)}^{\omega_{i}}={\prod_{i\;=\;1}^{m}\left(\frac{(1-\xi_{i}^{\prime })\eta_{i}^{\prime }}{\xi_{i}^{\prime }(1-\eta_{i}^{\prime })}\right)}^{\omega_{i}}\text{ and }{\prod_{i\;=\;1}^{m}\left(\frac{\xi_{i}\eta_{i}}{\left(1-\xi_{i})(1-\eta_{i}\right)}\right)}^{\omega_{i}}\geq {\prod_{i\;=\;1}^{m}\left(\frac{\xi_{i}^{\prime }\eta_{i}^{\prime }}{\left(1-\xi_{i}^{\prime })(1-\eta_{i}^{\prime }\right)}\right)}^{\omega_{i}}.$$

Let $\tilde{\alpha }=(\xi ,\eta )={\mathrm{ICRWG}}_{\omega }({\tilde{\alpha }}_{1},{\tilde{\alpha }}_{2},\ldots ,{\tilde{\alpha }}_{m})$, ${\tilde{\alpha }}^{\prime }=(\xi^{\prime },\eta^{\prime })={\mathrm{ICRWG}}_{\omega }({\tilde{\alpha }}_{1}^{\prime },{\tilde{\alpha }}_{2}^{\prime },\ldots ,{\tilde{\alpha }}_{m}^{\prime })$. By (10), one has $\frac{(1-\xi )\eta }{\xi (1-\eta )}={\prod_{i\;=\;1}^{m}\left(\frac{(1-\xi_{i})\eta_{i}}{\xi_{i}(1-\eta_{i})}\right)}^{\omega_{i}}<{\prod_{i\;=\;1}^{m}\left(\frac{(1-\xi_{i}^{\prime })\eta_{i}^{\prime }}{\xi_{i}^{\prime }(1-\eta_{i}^{\prime })}\right)}^{\omega_{i}}=\frac{(1-\xi^{\prime })\eta^{\prime }}{\xi^{\prime }(1-\eta^{\prime })}$ or

$\frac{(1-\xi )\eta }{\xi (1-\eta )}=\frac{(1-\xi^{\prime })\eta^{\prime }}{\xi^{\prime }(1-\eta^{\prime })}$ and

$\frac{\xi \eta }{\left(1-\xi )(1-\eta \right)}={\prod_{i\;=\;1}^{m}\left(\frac{\xi_{i}\eta_{i}}{\left(1-\xi_{i})(1-\eta_{i}\right)}\right)}^{\omega_{i}}\geq {\prod_{i\;=\;1}^{m}\left(\frac{\xi_{i}^{\prime }\eta_{i}^{\prime }}{\left(1-\xi_{i}^{\prime })(1-\eta_{i}^{\prime }\right)}\right)}^{\omega_{i}}=\frac{\xi^{\prime }\eta^{\prime }}{\left(1-\xi^{\prime })(1-\eta^{\prime }\right)}$.

Therefore, one can obtain $S(\tilde{\alpha })=\frac{\sqrt{\xi (1-\eta )}}{\sqrt{\xi (1-\eta )}-\sqrt{(1-\xi )\eta }}>\frac{\sqrt{\xi^{\prime }(1-\eta^{\prime })}}{\sqrt{\xi^{\prime }(1-\eta^{\prime })}-\sqrt{(1-\xi^{\prime })\eta^{\prime }}}=S({\tilde{\alpha }}^{\prime })$ or

$S(\tilde{\alpha })=S({\tilde{\alpha }}^{\prime })$ and $H(\tilde{\alpha })=\frac{\xi \eta }{\left(1-\xi )(1-\eta \right)}\geq \frac{\xi^{\prime }\eta^{\prime }}{\left(1-\xi^{\prime })(1-\eta^{\prime }\right)}=H({\tilde{\alpha }}^{\prime })$

This completes the proof of Theorem 6.

**Theorem 7.**  *(Idempotency). If* ${\tilde{\alpha }}_{i}=\tilde{\alpha }$ *(*i=1,2,…,m*), where* $\tilde{\alpha }\in \Theta$*, i.e., all A-IFVs* ${\tilde{\alpha }}_{i}$ (i=1,2,…,m*)* *are equal, then* ${\mathrm{ICRWG}}_{\omega }({\tilde{\alpha }}_{1},{\tilde{\alpha }}_{2},\ldots ,{\tilde{\alpha }}_{m})=\tilde{\alpha }$*.*

### 4.2. An ICRWG-Based Hierarchical Multicriteria Decision Model with Intuitionistic Preference Relations

The membership and non-membership values of an IPR $\tilde{R}={\left({\tilde{r}}_{ij}\right)}_{n\;\times \;n}={\left(\left(\xi_{ij},\eta_{ij}\right)\right)}_{n\;\times \;n}$ with $0<\xi_{ij},\eta_{ij}<1$ are provided by a DM based on an unbounded bipolar ]0, 1[-scale with the associated uninorm $\frac{xy}{xy+(1-x)(1-y)}$. This scale takes 0.5 as its neutral element to separate positive membership/non-membership judgments ξ>0.5/η>0.5 from negative ones ξ<0.5/η<0.5. Obviously, this scale is unbounded. We can see from Definition 5 that maximum and minimum A-IFVs do not exist in Θ. This makes it difficult to employ cross ratio based A-IFVs to express DMs’ pairwise judgments in practical decision making problems. To overcome this difficulty and better apply IPRs in decision modeling, a bounded and cross ratio based bipolar 0.1–0.9 scale is put forward as shown in the second column in Table 1.

If a judgment $\tilde{\alpha }=(\xi ,\eta )$ is given under the cross ratio based bipolar 0.1–0.9 scale, then $\tilde{\alpha }\in \Theta$ and 0.1≤ξ,η≤0.9,ξ+η≤1.

Let

(15) $$\Theta_{0.1-0.9}=\left\{(\xi ,\eta )|(\xi ,\eta )\in \Theta ,0.1\leq \xi ,\eta \leq 0.9\right\}$$

(16) $${ℜ}_{n\times n}^{0.1-0.9}=\left\{\tilde{R}|\tilde{R}={\left({\tilde{r}}_{ij}\right)}_{n\;\times \;n}={\left(\left(\xi_{ij},\eta_{ij}\right)\right)}_{n\;\times \;n},0.1\leq \xi_{ij},\eta_{ij}\leq 0.9,{\tilde{r}}_{ij}=(0.5,0.5),{\tilde{r}}_{ji}^{C}={\tilde{r}}_{ij},i,j=1,2,\ldots ,n\right\}$$

Then, by (4) and (7), we have $0.1\leq S(\tilde{\beta })\leq 0.9$ and $1/81\leq H(\tilde{\beta })\leq 1$ for any $\tilde{\beta }\in \Theta_{0.1-0.9}$. Moreover, S((0.1,0.9))=0.1,S((0.9,0.1))=0.9 and H((0.1,0.9))=H((0.9,0.1))=1. According to Definition 5, the A-IFVs (0.1, 0.9) and (0.9, 0.1) are the minimum and maximum elements in $\Theta_{0.1-0.9}$, respectively. Thus, the following corollary can be directly obtained from Theorem 5.

**Corollary 1.**  *Let* ${\tilde{\beta }}_{i}=(\xi_{i},\eta_{i})$ *(*i=1,2,…,m*) be m A-IFVs in* $\Theta_{0.1-0.9}$*, then* ${\mathrm{ICRWG}}_{\omega }({\tilde{\beta }}_{1},{\tilde{\beta }}_{2},\ldots ,{\tilde{\beta }}_{m})\in \Theta_{0.1-0.9}$ *and* $\left(0.9,0.1)\geq {\mathrm{ICRWG}}_{\omega }({\tilde{\beta }}_{1},{\tilde{\beta }}_{2},\ldots ,{\tilde{\beta }}_{m})\geq (0.1,0.9\right)$*.*

For any given IPR $\tilde{R}={\left({\tilde{r}}_{ij}\right)}_{n\;\times \;n}={\left(\left(\xi_{ij},\eta_{ij}\right)\right)}_{n\;\times \;n}\in {ℜ}_{n\;\times \;n}^{0.1-0.9}$, we can use the ICRWGM operator defined by (12) to aggregate the intuitionistic fuzzy judgments ${\tilde{r}}_{ij}$ (*j* = 1, 2, …, *n*) in the *i*th row of $\tilde{R}$ and obtain the priority of alternative $x_{i}$ (*i* = 1, 2, …, *n*), i.e., ${\tilde{\beta }}_{x_{i}}=\mathrm{ICRWGM}({\tilde{r}}_{i1}^{},{\tilde{r}}_{i2}^{},\ldots ,{\tilde{r}}_{in}^{})=\overset{n}{\underset{j\;=\;1}{⊗}}{\left({\tilde{r}}_{ij}^{}\right)}^{1/n}$. By Corollary 1, one has ${\tilde{\beta }}_{x_{i}}\in \Theta_{0.1-0.9}$ for all i=1,2,…,n.

Given the cross ratio based bipolar 0.1–0.9 scale and the proposed weighted geometric aggregation operator, a procedure is now developed for solving hierarchical decision problems with IPRs as follows.

**Procedure 1**  Step 1. Consider a two-level decision problem with a hierarchical structure, let $X=\{x_{1},x_{2},\ldots ,x_{n}\}$ be a set of alternatives, and $C=\{c_{1},c_{2},\ldots ,c_{m}\}$ be a group of criteria, whose importance weight vector is $\omega ={\left(\omega_{1},\omega_{2},\ldots ,\omega_{m}\right)}^{T}$ with $\sum_{l\;=\;1}^{m}\omega_{l}=1$ and $\omega_{l}\geq 0$ for all l=1,2,…,m. For each criterion $c_{l}$(l=1,2,…,m), a DM compares each pair of the alternatives in X under the cross ratio based bipolar 0.1–0.9 scale and furnishes his/her ratings by an IPR ${\tilde{R}}_{\left(l\right)}={\left({\tilde{r}}_{ij(l)}\right)}_{n\;\times \;n}={\left(\left(\xi_{ij(l)},\eta_{ij(l)}\right)\right)}_{n\;\times \;n}\in {ℜ}_{n\;\times \;n}^{0.1-0.9}$.

Step 2. For each IPR ${\tilde{R}}_{\left(l\right)}$(l=1,2,…,n), the ICRWGM operator defined by (12) is employed to aggregate the intuitionistic fuzzy judgments ${\tilde{r}}_{ij(l)}^{}$ (*j* = 1, 2, …, *n*) in the *i*th row of ${\tilde{R}}_{\left(l\right)}$. The local priority of alternative $x_{i}$ over criterion $c_{l}$ is derived as ${\tilde{\beta }}_{il}=\mathrm{ICRWGM}({\tilde{r}}_{i1(l)}^{},{\tilde{r}}_{i2(l)}^{},\ldots ,{\tilde{r}}_{in(l)}^{})=\overset{n}{\underset{j\;=\;1}{⊗}}{\left({\tilde{r}}_{ij(l)}^{}\right)}^{1/n}$ for all i=1,2,…,n,l=1,2,…,m.

Step 3. Construct an n×m decision matrix as $\tilde{B}={\left({\tilde{\beta }}_{il}\right)}_{n\;\times \;m}={\left(\left(\xi_{il},\eta_{il}\right)\right)}_{n\;\times \;m}$.

Step 4. Use the ICRWG operator (9) together with the criterion weight vector ω to fuse the local priorities ${\tilde{\beta }}_{il}$ (*l* = 1, 2, …, *m*) in the *i*th row of $\tilde{B}$ for each i=1,2,…,n, thereby obtaining the overall priority of alternative $x_{i}$ as

$$\begin{array}{l} {\tilde{\beta }}_{x_{i}}={\mathrm{ICRWG}}_{\omega }({\tilde{\beta }}_{i1},{\tilde{\beta }}_{i2},\ldots ,{\tilde{\beta }}_{im})=\overset{m}{\underset{l\;=\;1}{⊗}}{\left({\tilde{\beta }}_{il}\right)}^{\omega_{l}}\\ =\left(\frac{\prod_{l\;=\;1}^{m}{\left(\xi_{il}\right)}^{\omega_{l}}}{\prod_{l\;=\;1}^{m}{\left(\xi_{il}\right)}^{\omega_{l}}+\prod_{l\;=\;1}^{m}{\left(1-\xi_{il}\right)}^{\omega_{l}}},\frac{\prod_{l\;=\;1}^{m}{\left(\eta_{il}\right)}^{\omega_{il}}}{\prod_{l\;=\;1}^{m}{\left(\eta_{il}\right)}^{\omega_{l}}+\prod_{l\;=\;1}^{m}{\left(1-\eta_{il}\right)}^{\omega_{l}}}\right) \end{array}$$

Step 5. Calculate the score function value $S({\tilde{\beta }}_{x_{i}})$ and the accuracy function value $H({\tilde{\beta }}_{x_{i}})$ for each alternative $x_{i}$ (i=1,2,…,n) by employing (4) and (7).

Step 6. Rank alternatives or choose the best one(s) according to a decreasing order of ${\tilde{\beta }}_{x_{i}}$ (i=1,2,…,n) based on Definition 5.

## 5. A Case Study of Medical Waste Disposal Method Selection

This section applies the proposed decision procedure to a MWDMS problem in a medical institution in Hangzhou, China.

Rapid growth in global population and improved access to medical care have dramatically increased the amount of medical waste around the globe [2]. Improperly handled and disposed medical waste can pose a notable risk of infection or injury to health care personnel as well as a hazard to public health via micro-organism spreading [25]. As such, proper management and disposal of medical waste has become a daunting and demanding challenge for many jurisdictions. Generally speaking, medical waste cannot be disposed with the solid waste system owing to pathogenic considerations and there does not exist any disposal method that is environmental friendly and low cost [2]. Currently, the most commonly used disposal method is incineration, but this method arouses concerns about air pollution and combustion of medical waste may emit highly toxic substances such as dioxins, furans, and mercury. On the other hand, incineration has its advantage of ensuring sterilization and a significant reduction of waste volumes. Other options that are available for disposing medical waste include autoclave, microwave, sterilization, and other alternative treatment methods, and each one has its own advantages and disadvantages.

**Figure 1:**
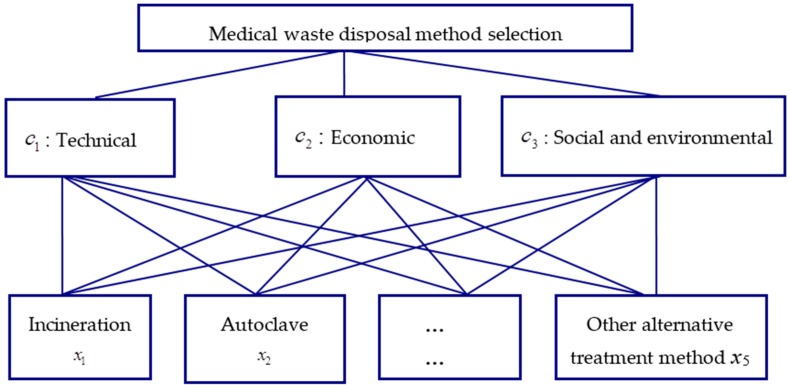
Decision hierarchy of a medical waste disposal method selection problem.

In this case study, five disposal methods are considered: incineration, autoclave, microwave, sterilization, and other alternative treatment methods, constituting the bottom alternative layer in the hierarchical decision structure in Figure 1. The middle layer furnishes the three evaluation criteria for the alternatives, consisting of technical, economic, and social and environmental considerations. The very top of Figure 1 signifies that the overall objective of this decision structure is to assist the selection of the best method to dispose medical waste.

First, at the bottom, pairwise comparisons of the five methods are conducted under each criterion and these ratings are furnished as three IPRs ${\tilde{R}}_{\left(l\right)}={\left({\tilde{r}}_{ij(l)}\right)}_{5\;\times \;5}=$ ${\left(\left(\xi_{ij(l)},\eta_{ij(l)}\right)\right)}_{5\;\times \;5}$, *l* = 1, 2, 3, under the cross ratio based bipolar 0.1–0.9 scale. Table 2, Table 3 and Table 4 display the DM’s pairwise comparison of the five disposal methods under the three criteria, technical, economic, and social and environmental, respectively. In these three judgment matrices, one can observe that all of the diagonal elements are (0.5, 0.5), indicating that any disposal method is indifferent to itself. The other elements reflect the DM’s pairwise judgment for one disposal method over the other in terms of an A-IFV assessment in a cross ratio based bipolar 0.1–0.9 scale under different evaluation criteria. For instance, the cell at the intersection of row *x*_1_ and column *x*_2_ in Table 2 gives an A-IFV (5/8, 1/5), indicating the DM’s preference (from a technical perspective) of *x*_1_ (incineration) to *x*_2_ (autoclave) as 5/8 and its preference of *x*_2_ (autoclave) to *x*_1_ (incineration) as 1/5 under the cross ratio based bipolar 0.1–0.9 scale. The other values in Table 2 represent the DM’s assessment of other pairwise comparisons from the technical consideration. In a similar fashion, one can interpret the non-diagonal elements in Table 3 and Table 4 as the DM’s pairwise judgments of the five disposal methods under the other two criteria (economic and social and environmental). By balancing the considerations from the technical, economic, and social and environmental aspects, the DM provides his/her weight vector for the three criteria as $\omega ={\left(\omega_{1},\omega_{2},\omega_{3}\right)}^{T}=$ ${\left(0.4,0.35,0.25\right)}^{T}$.

For each IPR ${\tilde{R}}_{\left(l\right)}$(l=1,2,3), we employ the ICRWGM operator to fuse judgments in the *i*th row of ${\tilde{R}}_{\left(l\right)}$ and derive local priorities as shown in the (*l* + 1)th column in Table 5 for each alternative under criterion $c_{l}$.

Based on the decision matrix $\tilde{B}$ listed in Table 5, we use ICRWG operator (9) together with the aforesaid criterion weight vector ω to aggregate the local priorities ${\tilde{\beta }}_{il}$ (*l* = 1, 2, 3) in the *i*th row of $\tilde{B}$ for each i=1,2,…,5, and obtain the overall priorities expressed as A-IFVs ${\tilde{\beta }}_{x_{i}}$ (i=1,2,…,5) for the five alternatives.

${\tilde{\beta }}_{x_{1}}=(0.6930,0.2042)$, ${\tilde{\beta }}_{x_{2}}=(0.4867,0.3819)$, ${\tilde{\beta }}_{x_{3}}=(0.3321,0.5640)$, ${\tilde{\beta }}_{x_{4}}=(0.2933,0.6217)$, ${\tilde{\beta }}_{x_{5}}=(0.3980,0.4642)$.

According to the score function (4), one has $S\left({\tilde{\beta }}_{x_{1}}\right)=0.7479$, $S\left({\tilde{\beta }}_{x_{2}}\right)=0.5533$, $S\left({\tilde{\beta }}_{x_{3}}\right)=0.3827$, $S\left({\tilde{\beta }}_{x_{4}}\right)=0.3345$ and $S\left({\tilde{\beta }}_{x_{5}}\right)=0.4664$.

As $S\left({\tilde{\beta }}_{x_{1}}\right)>S\left({\tilde{\beta }}_{x_{2}}\right)>S\left({\tilde{\beta }}_{x_{5}}\right)>S\left({\tilde{\beta }}_{x_{3}}\right)>S\left({\tilde{\beta }}_{x_{4}}\right)$, the ranking of the five medical waste disposal methods is derived as $x_{1}>x_{2}>x_{5}>x_{3}>x_{4}$. Although many concerns have been raised about the incineration method, it remains the most viable option to safely dispose medical waste. For instance, incineration has accounted for about 49%–60% of medical waste in the USA [2].

## 6. Conclusions

In this paper, a two-level hierarchical multicriteria decision model is proposed to assist in the selection of the best medical waste disposal method, where criteria weights are known and pairwise comparison results are expressed as A-IFVs in a cross ratio based bipolar 0.1–0.9 scale. To facilitate processing decision information in A-IFVs, we first propose new operational laws for ]0, 1[-valued A-IFVs and put forward their desirable properties. Score and accuracy functions are then introduced for devising a comparison approach for A-IFVs. We subsequently develop an ICRWG operator for aggregating ]0, 1[-valued A-IFVs. To effectively apply A-IFVs to preference modeling, we next present a cross ratio based bipolar 0.1–0.9 scale and formulate a procedure for solving hierarchical multicriteria decision problems with IPRs. This decision framework is then applied to a MWDMS case study to illustrate how it can be applied in practice.

## Figures and Tables

**Table 1:** Cross ratio based bipolar 0.1–0.9 scale.

Saaty’s 1–9 Scale	Ratio Based Bipolar 0.1–0.9 Scale	Meaning
9	0.9	Absolutely preferred
7	7/8	Very strongly preferred
5	5/6	Strongly preferred
3	3/4	Moderately preferred
1	0.5	Equally preferred
1/3	1/4	Moderately less preferred
1/5	1/6	Strongly less preferred
1/7	1/8	Very strongly less preferred
1/9	0.1	Extremely less preferred
Other values r between 1/9 and 9	Other values r/(1+r) between 0.1 and 0.9	In-between values for intermediate preference

**Table 2:** Intuitionistic preference relation ${\tilde{R}}_{\left(1\right)}={\left({\tilde{r}}_{ij(1)}\right)}_{5\;\times \;5}={\left(\left(\xi_{ij(1)},\eta_{ij(1)}\right)\right)}_{5\;\times \;5}$.

	$x_{1}$	$x_{2}$	$x_{3}$	$x_{4}$	$x_{5}$
$x_{1}$	(0.5, 0.5)	(5/8, 1/5)	(7/8, 0.1)	(3/4, 1/8)	(0.5, 1/6)
$x_{2}$	(1/5, 5/8)	(0.5, 0.5)	(5/8, 1/5)	(2/3, 1/6)	(3/8, 0.5)
$x_{3}$	(0.1, 7/8)	(1/5, 5/8)	(0.5, 0.5)	(0.5, 3/8)	(1/5, 5/8)
$x_{4}$	(1/8, 3/4)	(1/6, 2/3)	(3/8, 0.5)	(0.5, 0.5)	(3/7, 3/7)
$x_{5}$	(1/6, 0.5)	(0.5, 3/8)	(5/8, 1/5)	(3/7, 3/7)	(0.5, 0.5)

**Table 3:** Intuitionistic preference relation ${\tilde{R}}_{\left(2\right)}={\left({\tilde{r}}_{ij(2)}\right)}_{5\;\times \;5}={\left(\left(\xi_{ij(2)},\eta_{ij(2)}\right)\right)}_{5\;\times \;5}$.

	$x_{1}$	$x_{2}$	$x_{3}$	$x_{4}$	$x_{5}$
$x_{1}$	(0.5, 0.5)	(3/4, 1/8)	(6/7, 0.1)	(7/8, 0.1)	(5/8, 1/5)
$x_{2}$	(1/8, 3/4)	(0.5, 0.5)	(3/4, 1/8)	(5/8, 1/4)	(3/7, 3/7)
$x_{3}$	(0.1, 6/7)	(1/8, 3/4)	(0.5, 0.5)	(6/7, 1/8)	(3/8, 0.5)
$x_{4}$	(0.1, 7/8)	(1/4, 5/8)	(1/8, 6/7)	(0.5, 0.5)	(4/7, 3/8)
$x_{5}$	(1/5, 5/8)	(3/7, 3/7)	(0.5, 3/8)	(3/8, 4/7)	(0.5, 0.5)

**Table 4:** Intuitionistic preference relation ${\tilde{R}}_{\left(3\right)}={\left({\tilde{r}}_{ij(3)}\right)}_{5\;\times \;5}={\left(\left(\xi_{ij(3)},\eta_{ij(3)}\right)\right)}_{5\;\times \;5}$.

	$x_{1}$	$x_{2}$	$x_{3}$	$x_{4}$	$x_{5}$
$x_{1}$	(0.5, 0.5)	(3/5, 1/3)	(2/3, 1/4)	(3/4, 1/5)	(5/7, 1/7)
$x_{2}$	(1/3, 3/5)	(0.5, 0.5)	(3/5, 2/7)	(5/7, 1/4)	(4/7, 2/7)
$x_{3}$	(1/4, 2/3)	(2/7, 3/5)	(0.5, 0.5)	(4/7, 2/7)	(3/7, 3/7)
$x_{4}$	(1/5, 3/4)	(1/4, 5/7)	(2/7, 4/7)	(0.5, 0.5)	(3/7, 0.5)
$x_{5}$	(1/7, 5/7)	(2/7, 4/7)	(3/7, 3/7)	(0.5, 3/7)	(0.5, 0.5)

**Table 5:** Decision matrix $\tilde{B}={\left({\tilde{\beta }}_{il}\right)}_{5\;\times \;3}={\left(\left(\xi_{il},\eta_{il}\right)\right)}_{5\;\times \;3}$.

	$c_{1}$	$c_{2}$	$c_{3}$
$x_{1}$	(0.6706, 0.1934)	(0.7445, 0.1758)	(0.6508, 0.2701)
$x_{2}$	(0.4654, 0.3783)	(0.4688, 0.3901)	(0.5457, 0.3763)
$x_{3}$	(0.2701, 0.6204)	(0.3607, 0.5471)	(0.4006, 0.4947)
$x_{4}$	(0.2951, 0.5746)	(0.2708, 0.6786)	(0.3235, 0.6131)
$x_{5}$	(0.4311, 0.3925)	(0.3925, 0.5000)	(0.3545, 0.5314)
